# Germline Transgenesis and Insertional Mutagenesis in *Schistosoma mansoni* Mediated by Murine Leukemia Virus

**DOI:** 10.1371/journal.ppat.1002820

**Published:** 2012-07-26

**Authors:** Gabriel Rinaldi, Sabine E. Eckert, Isheng J. Tsai, Sutas Suttiprapa, Kristine J. Kines, José F. Tort, Victoria H. Mann, Daniel J. Turner, Matthew Berriman, Paul J. Brindley

**Affiliations:** 1 Department of Microbiology, Immunology & Tropical Medicine, School of Medicine & Health Sciences, The George Washington University, Washington, DC, United States of America; 2 Departamento de Genética, Facultad de Medicina, Universidad de la República, (UDELAR), Montevideo, Uruguay; 3 The Wellcome Trust Sanger Institute, Wellcome Trust Genome Campus, Hinxton, Cambridge, United Kingdom; 4 Oxford Nanopore Technologies, Oxford, United Kingdom; 5 Department of Pathobiology, Faculty of Science, Mahidol University, Bangkok, Thailand; 6 Tulane Cancer Center, Tulane University Health Sciences Center, New Orleans, Louisiana, United States of America; 7 Research Center for Neglected Diseases of Poverty, The George Washington University, Washington, DC, United States of America; Institute of Molecular Genetics, Czech Republic

## Abstract

Functional studies will facilitate characterization of role and essentiality of newly available genome sequences of the human schistosomes, *Schistosoma mansoni*, S. *japonicum* and *S. haematobium*. To develop transgenesis as a functional approach for these pathogens, we previously demonstrated that pseudotyped murine leukemia virus (MLV) can transduce schistosomes leading to chromosomal integration of reporter transgenes and short hairpin RNA cassettes. Here we investigated vertical transmission of transgenes through the developmental cycle of *S. mansoni* after introducing transgenes into eggs. Although MLV infection of schistosome eggs from mouse livers was efficient in terms of snail infectivity, >10-fold higher transgene copy numbers were detected in cercariae derived from *in vitro* laid eggs (IVLE). After infecting snails with miracidia from eggs transduced by MLV, sequencing of genomic DNA from cercariae released from the snails also revealed the presence of transgenes, demonstrating that transgenes had been transmitted through the asexual developmental cycle, and thereby confirming germline transgenesis. High-throughput sequencing of genomic DNA from schistosome populations exposed to MLV mapped widespread and random insertion of transgenes throughout the genome, along each of the autosomes and sex chromosomes, validating the utility of this approach for insertional mutagenesis. In addition, the germline-transmitted transgene encoding neomycin phosphotransferase rescued cultured schistosomules from toxicity of the antibiotic G418, and PCR analysis of eggs resulting from sexual reproduction of the transgenic worms in mice confirmed that retroviral transgenes were transmitted to the next (F1) generation. These findings provide the first description of wide-scale, random insertional mutagenesis of chromosomes and of germline transmission of a transgene in schistosomes. Transgenic lines of schistosomes expressing antibiotic resistance could advance functional genomics for these significant human pathogens.

**Database accession:**

Sequence data from this study have been submitted to the European Nucleotide Archive (http://www.ebi.ac.uk/embl) under accession number ERP000379.

## Author Summary

Schistosomes, or blood flukes, are responsible for the major neglected tropical disease called schistosomiasis, which afflicts over 200 million people in impoverished regions of the developing world. The genome sequence of these parasites has been decoded. Integration sites of retroviral transgenes into the chromosomes of schistosomes were investigated by high-throughput sequencing. Transgene integrations were mapped to the genome sequence of *Schistosoma mansoni*. Integrations were distributed apparently randomly across each of the eight chromosomes, including the seven autosomes and the sex chromosomes Z and W. Integration events of transgenes were characterized in chromosomes of cercariae that were progeny of schistosome eggs infected with pseudotyped virions. Also, transgenic cercariae were employed to infect mice and transgenes were detected in the F1 eggs. Together these findings confirmed vertical transmission of transgenes through the schistosome germline, through both the asexual and the sexual reproductive phases of the developmental cycle. Moreover, germline-transmitted retroviral transgenes encoding drug resistance to the aminoglycoside antibiotics allowed schistosomes to survive toxic concentrations of the antibiotic G418. These findings represent the first reports of wide-scale insertional mutagenesis of schistosome chromosomes and vertical transmission of a transgene through the schistosome germline.

## Introduction

The schistosomes are considered the most important helminth pathogens in terms of human morbidity and mortality. More than 200 million people are infected and a further 800 million at risk of schistosomiasis in tropical and sub-tropical latitudes. Treatment and control of schistosomiasis rely on the anthelmintic drug praziquantel; however, there is concern that drug resistance will develop. New therapeutic approaches including vaccines, drugs and diagnostics are needed for this neglected tropical disease [1]–[6]. Through a complex two-host life cycle, schistosomes are transmitted from freshwater snails to humans. Adult schistosomes dwell as pairs in the blood vessels of the intestines and/or urinary bladder, where female worms release eggs that become embedded in the intestinal wall and other organs to elicit chronic immune-mediated disease and other serious complications [7].

Draft genome sequences for *Schistosoma japonicum*, *S. mansoni* and *S. haematobium* were reported recently, landmark events that ushered in the post-genomic era for schistosomiasis [8]–[11]. In brief, the haploid genome size of these blood flukes is 364–397 MB; they have eight pairs of chromosomes, seven autosomes and a pair of sex chromosomes Z and W bearing ∼11,000 protein-encoding genes, the genome is >60% AT, and 40–50% of the genome is constituted of repetitive and mobile elements. In addition to extensive genomic and transcriptomic datasets, functional analysis of target genes to underpin new interventions for schistosomiasis will require both reverse and forward genetics [10]. To date, functional genomics beyond conventional RNA interference have not generally been available for schistosomes (e.g. see [12]–[14]). Nonetheless, reporter plasmids and RNAs have been introduced to several developmental stages [5], [15]–[22]. Moreover, the *piggyBac* transposon has been shown to competently integrate into schistosome chromosomes [23] and germline transmission of extrachromosomal, plasmid transgenes through several generations has been reported [15]. Development of somatic and germline transgenesis for schistosomes can be expected to facilitate validation of essential genes/gene products to be targeted with drugs or vaccines, as attested by progress with other pathogens e.g. *Plasmodium falciparum* [24], *Toxoplasma gondii* [25], *Candida albicans* [26] and *Salmonella enterica* serovar Typhi [27].

Recently, it has been demonstrated that pseudotyped murine leukemia virus (MLV), widely used in human gene therapy e.g. [28], can be adapted for genetic transformation of schistosomes. Reporter transgenes can be introduced and expressed; gain-of-function, including expression of firefly luciferase and antibiotic selection [29] and loss-of-function through vector based RNA interference has been achieved [30]–[32]. Here we used MLV for insertional mutagenesis of schistosome chromosomes and investigated target site specificity of integrated MLV retrovirus, employing high throughput sequencing approaches and a revised schistosome genome sequence. In addition, by characterizing integration events in schistosomes that had been exposed to the pseudotyped virions as eggs, we determined that the retroviral genes were transmitted through the germline. In addition, mice were infected by the percutaneous route with transgenic cercariae, after which transgenes were detected in F1 generation eggs. These findings represent the first report of wide-scale insertional mutagenesis of schistosome chromosomes and the first report of vertical, germline transmission of an integrated transgene in schistosomes. Moreover, they indicate how transgenic schistosomes, for example by expressing antibiotic resistance, could advance functional genomics for these neglected tropical disease pathogens.

## Results

### Retroviral transduction of schistosome eggs facilitates germ line transmission of transgenes

**Figure 1:**
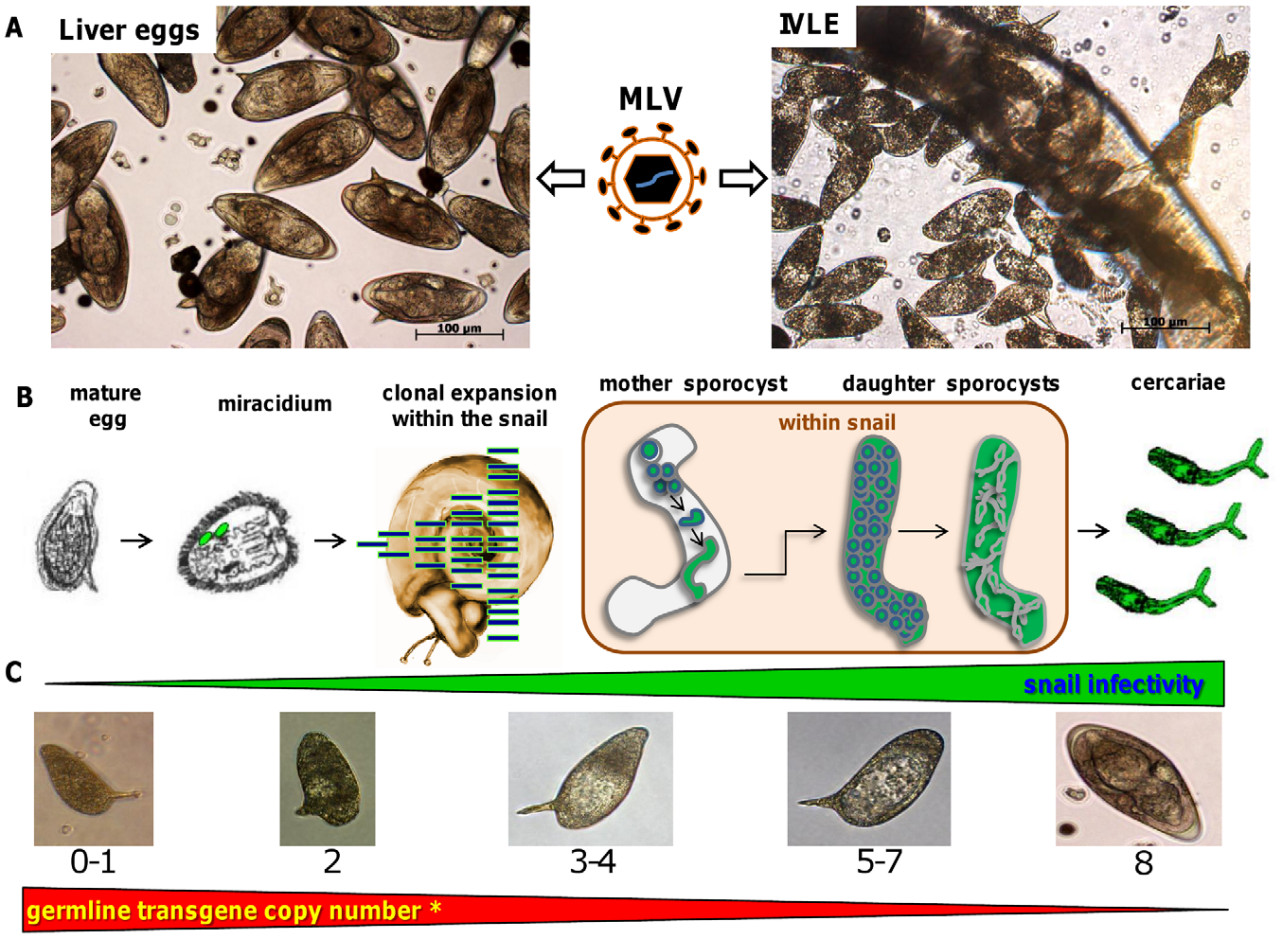
Schematic overview of approaches employed to introduce retroviral transgenes into the germline of *Schistosoma mansoni*. A: Left panel, micrograph showing viable schistosome eggs recovered from livers of *S. mansoni*-infected mice. Center panel, schematic representation of the MLV virion inoculated into the culture media. Right panel, micrograph of eggs laid *in vitro* by cultured mix sexed adults of *S. mansoni*; B: outline of germline transmission in *S. mansoni*. From the single-celled zygote in the newly fertilized egg that is released by the female schistosome, germ cells are propagated through the intra-snail developmental stages. Within the snail, daughter sporocysts arise from the germ cells of the mother sporocyst, and eventually cercariae develop from the daughter sporocysts. C: Representative micrographs of the embryogenesis of the *S. mansoni* egg; numbers below the images indicate the embryonic stage according to the staging system of Jurberg et al [42]. The zygotic stage (also termed stage 0) occurs inside the female worm. In culture, development from stages 0 to 8 takes about one week. Triangles above (green) and below (red) the eggs indicate the efficiency of snail infection and transgene copy number using transgenesis approaches targeting LE and IVLE, respectively. In regard to the green triangle, IVLE were cultured for seven days, until stage 8, before they were induced to hatch. *Pertaining to the red triangle, transgene copy numbers were measured in genomic DNA from cercariae.

Vertical or germ line transmission of transgenes mediated by integration competent vectors has not been reported in schistosomes. In order to establish a method of inserting retroviral transgenes in the schistosome germline, we targeted two populations of the schistosome egg. First, eggs were isolated from livers of experimentally infected mice (here termed ‘LE’, ‘liver eggs’), using standard approaches (Figure 1A, left panel). Second, *in vitro* laid eggs (IVLE), released from cultured schistosomes from 0 to 48 hours after perfusion of the adult worms from experimentally infected mice [33], [34] were collected (Figure 1A, right panel). Eggs derived by both methods were exposed to pseudotyped MLV virions (similar viral titers used in both methods) and the IVLE cultured to maturity (Figure 1A, center panel). Mature eggs were transferred to water, after which some hatched releasing miracidia. Immature *Biomphalaria glabrata* snails (≤5 mm diameter) were exposed to these miracidia (Table 1). Infected snails were maintained in the laboratory at 24°C for >40 days, then exposed to bright light to induce release of cercariae (Figure 1B). Genomic DNA was isolated from cercariae released from these snails, and transgene copy number and integration sites were investigated.

**Table 1:** Establishing germline transgenesis by retroviral transduction of *Schistosoma mansoni* eggs and infection of *Biomphalaria glabrata* snails with miracidia from virion transduced eggs.

A LIVER EGGS (LE)						
Experiment	MLV construct	MLV delivery	Miracidia per snail	Exposed snails	Shed	Transgene copies/nanogram of DNA
1	SmAct-Luc	S	≤10	14	+	0.5
2	SmAct-Luc	S	≤30	14	+	69.7
3	SmAct-Luc	S	≤100	14	+	1.8
4	SmAct-Luc	E	≤70	25	+	0*
5	SmAct-Luc	S	100	25	+	4.4*
6	SmAct-Luc	E	200	25	+	3.4*
7	SmAct-Luc	S	200	25	+	7.4*
8	SmAct-Luc	E	120	25	+	0**
9	SmAct-Luc	S	40	25	+	9**
10	SmAct-Luc	E	180	25	+	0.3***
11	SmAct-Luc	S	180	25	+	0***
12	SmAct-Luc	S	≤200	16	+	13∧
13	pLNHX-delta	S	100	25	+	316∧∧

B *IN VITRO* LAID EGGS (IVLE)						
1	pLNHX-basic	S	ND	25	−	NA
2	pLNHX-basic	S	ND	10	−	NA
3	pLNHX-basic	S	ND	10	+	0
4	No virions	S	ND	10	+	0
5	No virions	S	ND	10	−	NA
6	SmAct-Luc	S	ND	5	−	NA
7	No virions	S	ND	5	−	NA
8	No virions	S	ND	5	−	NA
9	SmAct-Luc	S	ND	6	+	2,057/479¶ ^,^ ∧∧ ^,^ †
10	pLNHX-delta	S	ND	5	−	NA
11	pLNHX-delta	S	ND	5–10	+	767
12	pLNHX-cHS4	S	ND	5–10	+	346
13	pLNHX-delta	S	ND	5–10	−	NA
14	pLNHX-cHS4	S	ND	5–10	−	NA

Outcomes of replicate experiments are presented in terms of release of cercariae from snails and transgene copy numbers per nanogram of genomic DNA isolated from cercariae are presented. Eggs from (A) livers of infected mice (LIVER EGGS) or (B) and *IN VITRO* LAID EGGS (IVLE) were exposed to pseudotyped murine leukemia virus (MLV) virions, hatched, and snails were exposed to the resulting miracidia.

*: , average of 3 sheds;

**: , average of 7 sheds;

***: , average of 2 sheds;

**∧:** , average of 4 sheds. Copy numbers/ng of gDNA of four consecutive sheds were 33, 15, 3 and 0;

**¶:** , 2057 was from three snails, 479 was from the one of these three snails that survived longest;

**∧∧:** , subjected to Illumina sequencing;

**†:** cercariae were GO generation of transgenic line named IVLE_MLV_001; ND, not determined; NA, not applicable; S, soaking; E, electroporation.

Twenty seven independent infections (transductions) of schistosome eggs were carried out, 13 on LE, and 14 on IVLE. Virion-free mock infections were performed to determine the viability of the two different egg types. Several prominent trends were seen. In each of 13 transductions of LE, cercariae were shed from infected snails, indicating the high infectivity of miracidia. By contrast, in only five of 14 (36%) experiments on IVLE were snails productively infected with miracidia leading eventually to release of cercariae. In addition, transgene copy numbers determined by qPCR were higher in the cercariae derived from IVLE than LE with a mean of 634 copies/ng of genomic DNA (range, 0–2,057) versus 33 copies/ng of genomic DNA (0–316), respectively (Table 1; Figure 1). In overview, miracidia from LE were more viable and infectious, reflecting higher overall fitness, than those from IVLE, but cercariae originating from IVLE carried a higher chromosomal density of transgenes (by at least one order of magnitude) (Figure 1C).

In addition, in initial investigation of the longevity of propagated transgenes, F1 progeny eggs from one of the lines originating from virion transduced IVLE (Table 1, IVLE experiment 9, the origin of the transgenic line named IVLE_MLV_001) were examined. Transgenic cercariae were employed to infect mice by the percutaneous route and, after 42 days, mouse feces examined for schistosome eggs. Eggs were seen from day 55 onwards (Figure S1, panel A). At day 65, the mice were euthanized and adult worms perfused from the portal system. IVLE (F1) were collected from the adult schistosomes (G0) (Figure S1, panels B, C). The *luciferase* transgene was detected by end-point PCR in adult worms (G0) (Figure S1, D) and by qPCR in IVLE, i.e. the F1 generation (Figure S1, E); the *neoR* transgene was also detected in the F1 eggs (not shown).

We also investigated the use of electroporation to transduce eggs with MLV. LE were exposed to MLV by square wave electroporation. In addition, we examined release of cercariae from individual snails rather from populations of infected snails. Higher transgene copy numbers were seen in cercariae from snails infected with miracidia from eggs soaked with MLV, whereas lower copy numbers were seen in cercariae derived from the eggs subjected to electroporation (e.g., Table 1A, experiment number 6 versus 12). The transgene copy number detected in cercariae released from the same group of snails varied from day to day (not shown). Cercariae from individual snails exhibited variable numbers of transgenes indicating that, among batches of infected snails, not all snails were parasitized by transgenic sporocysts (not shown).

### MLV integrates widely and randomly throughout schistosome chromosomes

**Figure 2:**
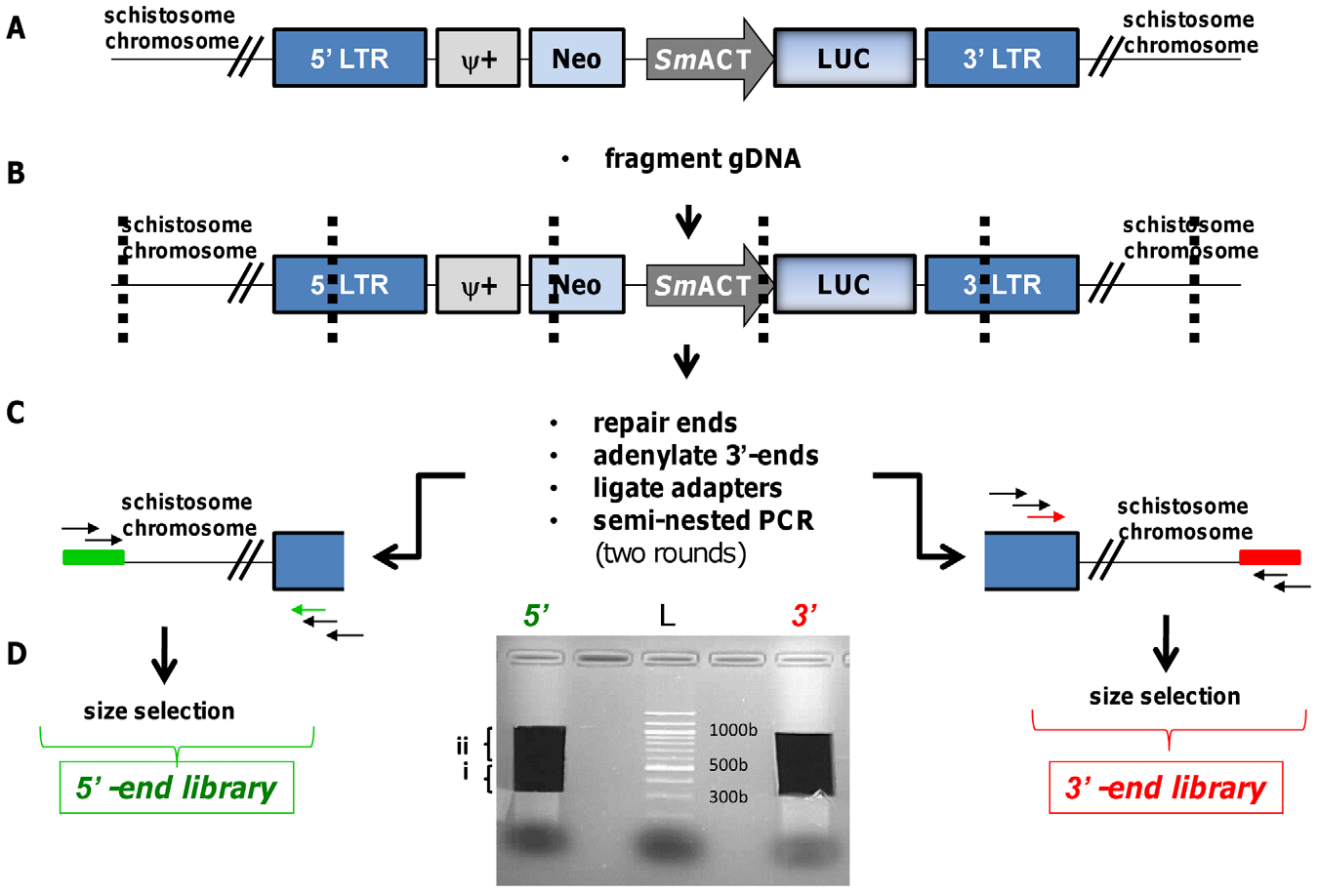
Construction of Illumina libraries from virion transduced schistosomes. A: Schematic representation of a representative MLV retrovirus construct integrated into the gDNA isolated from MLV-transduced organisms. The retrovirus cassette included the firefly luciferase reporter gene (LUC) driven by the *S. mansoni* actin 1.1 gene promoter (*Sm*ACT), and flanked by the 5′- and 3′-long terminal inverted repeats of the murine leukemia virus (LTR). The cassette also included the gene endowing neomycin resistance (*neoR*) and the psi motif (ψ) involved in packaging the viral DNA; B: Mechanical fragmentation of the genomic DNA; C: Repair of the fragment ends, adenylation, ligation of the Illumina adapters, and two rounds of semi-nested PCR (colored primers represent the sequencing primers); D: Size selection of the 5′-end (5′) and 3′-end (3′) libraries and gel extraction. The fragment selected from 300 bp to 500 bp (i) was employed to generate the libraries. A higher fragment (ii) was purified and stored as back up. The gel extracted and purified libraries were quantified by qPCR and loaded into Illumina flowcells. L, molecular size standards (ladder).

Genomic DNA (gDNA) was isolated from populations of schistosomules, adult worms and/or cercariae. Using sonication to fragment the genomic DNA, along with ligation of linkers to the fragments, over fifty Illumina libraries of PCR products amplified to enrich sites of retroviral integrations into the schistosome chromosomes were prepared. Subsequently, high throughput sequencing of the PCR products was undertaken (Figure 2). 5,556,734 paired sequence reads with retroviral start sites were obtained from 50 libraries constructed from both the 5′- and 3′-long terminal repeats (LTRs) of MLV from populations of adults and schistosomules exposed to virions (somatic transgenesis approach), and from cercariae that were the progeny of schistosome eggs exposed to virions (germline transgenesis approach). Sites of integrations of MLV provirus into schistosome chromosomes were predicted using stringent criteria and version 5.1 of the genome of *S. mansoni* [35]. 1,248 non-redundant events were located, 343 of which were obtained from both 5′- and 3′-LTR libraries from the somatic transgenesis approaches with schistosomules and adults, and 905 from cercarial gDNA libraries from the germline transgenesis (Table 2). Interestingly, three of the integrations included both 5′- and 3′- sequences.

**Table 2:** Locations of MLV retroviral transgene integrations within the genome of *Schistosoma mansoni*.

Integration count						
Genomic feature	Genome* (%)	Percentage of integration sites (%)				
		Somatic		Germline		Total
		5′	3′	5′	3′	
**Exons**	4.2	3.5	5.0	6.3	4.9	5.1
**Introns**	39.9	35.1	40.0	38.7	42.0	39.7
**Promoters/5′-UTR regions**	8.8	6.9	5.0	5.8	7.5	6.7
**Intergenic regions**	47.1	54.5	50.0	49.2	45.6	48.5
*Chi-square p-values*		*0.51*	*0.75*	*0.21*	*0.7*	*0.14*

*: proportion of sequence content.

‘Somatic’ refers to schistosomes (adult or schistosomulum stages) transduced directly with pseudotyped MLV virions. ‘Germline’ refers to the schistosomula derived as progeny from schistosome eggs transduced with virions. Somatic 5′, Somatic 3′, etc. refer to Illumina libraries constructed from regions of the schistosome genome flanking the 5′- or 3′-LTR of the retrovirus.

‘Promoter/5′-UTR regions’, ≤3 kb upstream of first exon of genes.

**Figure 3:**
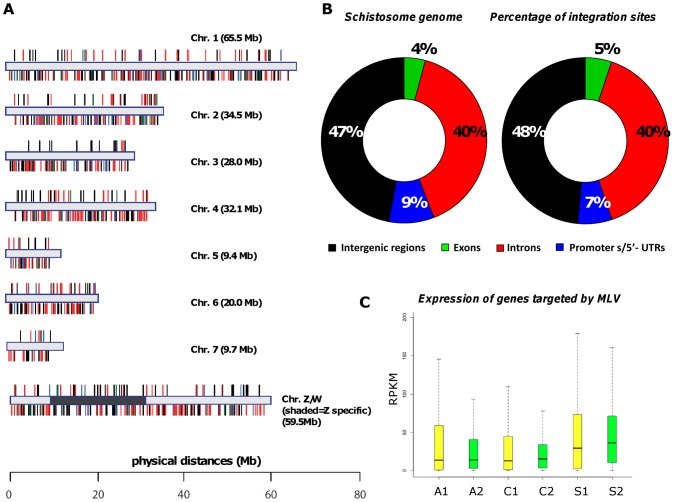
Integration events within the genome of MLV-transduced *Schistosoma mansoni*. Panel A: Horizontal bars represent the chromosomes of *S. mansoni*. MLV integration sites shown above the chromosomes were detected in genomic DNA from transduced adults and schistosomules (somatic transgenesis approach). MLV integration sites shown below the chromosomes were detected in genomic DNA from cercariae derived from MLV-transduced eggs/miracidia (germline transgenesis approach). Colors of integration sites follow the color code indicated in panel B. The scale bar indicates physical distances of the map. Panel B: Left, pie chart showing the percentages of the indicated regions in the genome of *S. mansoni*. Right, pie chart showing the percentages of MLV integration sites classified according to the function of the mapping site; Panel C: Box plots showing expression levels of genes carrying MLV transgenes (green) and transcriptomes at large (yellow) of the indicated developmental stages. A1, wild-type adults; A2, transgenic adults; C1, wild-type cercariae; C2, transgenic cercariae; S1, wild-type schistosomules; S2, transgenic schistosomules. RPKM, reads per kilobase per million mapped reads.

Chromosomal proviral integration sites are illustrated schematically in Figure 3. Integration events occurred broadly and seemingly randomly across each of the schistosome autosomes and both the Z and W sex chromosomes, including into Z-specific regions (Figure 3 A). For each integration event, the site of integration was characterized as intergenic, promoter/5′-UTR (up to 3 kb of the genome upstream of the first exon), exonic or intronic. This analysis revealed that 48.6% of the integrations occurred in intergenic regions, 39.7% in introns of coding regions, 5.1% of the MLV integrations were found in exons, and 6.7% in promoter/5′-UTRs (Table 2 and Figure 3 B). By comparison, 47% of the *S. mansoni* genome is composed of intergenic sequences, exons, 4.1%, promoter/5′-UTRs, 8.8% and introns 40% of the genome (Figure 3B, left). Table 2 presents details of integrations for 5′ and 3′ libraries from somatic and germline experiments. Comparison of the four functional categories of the genome by Chi-square analysis among the transgenic genome and non-transgenic reference revealed no significant differences. Using recent RNA Seq findings and database [35], levels of expression of genes carrying transgenes were compared to the transcriptomes at large of the MLV transduced developmental stages. No bias was evident for MLV to integrate into genes predicted to be actively transcribed at the developmental stage during viral transduction (Figure 3C).

### Proviral transgenes are generally intact

We had noted deletions in proviral transgenes in schistosome chromosomes in an earlier study [36]. To examine the extent and potential impact of this phenomenon in the current high throughput analysis, we constructed several libraries of the somatic genomic DNAs employing retroviral specific primers that targeted sites at 0, 500, 1,000 and 1,500 bp from the terminus of the retrovirus (Table S1, Figure S2). Integration events were determined in each of the eight library categories, demonstrating truncation of proviral transgenes. Moreover, integrations were located in all library categories, showing that truncations occurred at sites ranging from the 5′- or 3′-termini to as far as 1,500 bp from the terminus (Figure S3). Overall, 75% of the integrations were intact, whereas 25% of integrations exhibited truncations.

### Vertical transgene transmission confers antibiotic resistance to schistosomes

**Figure 4:**
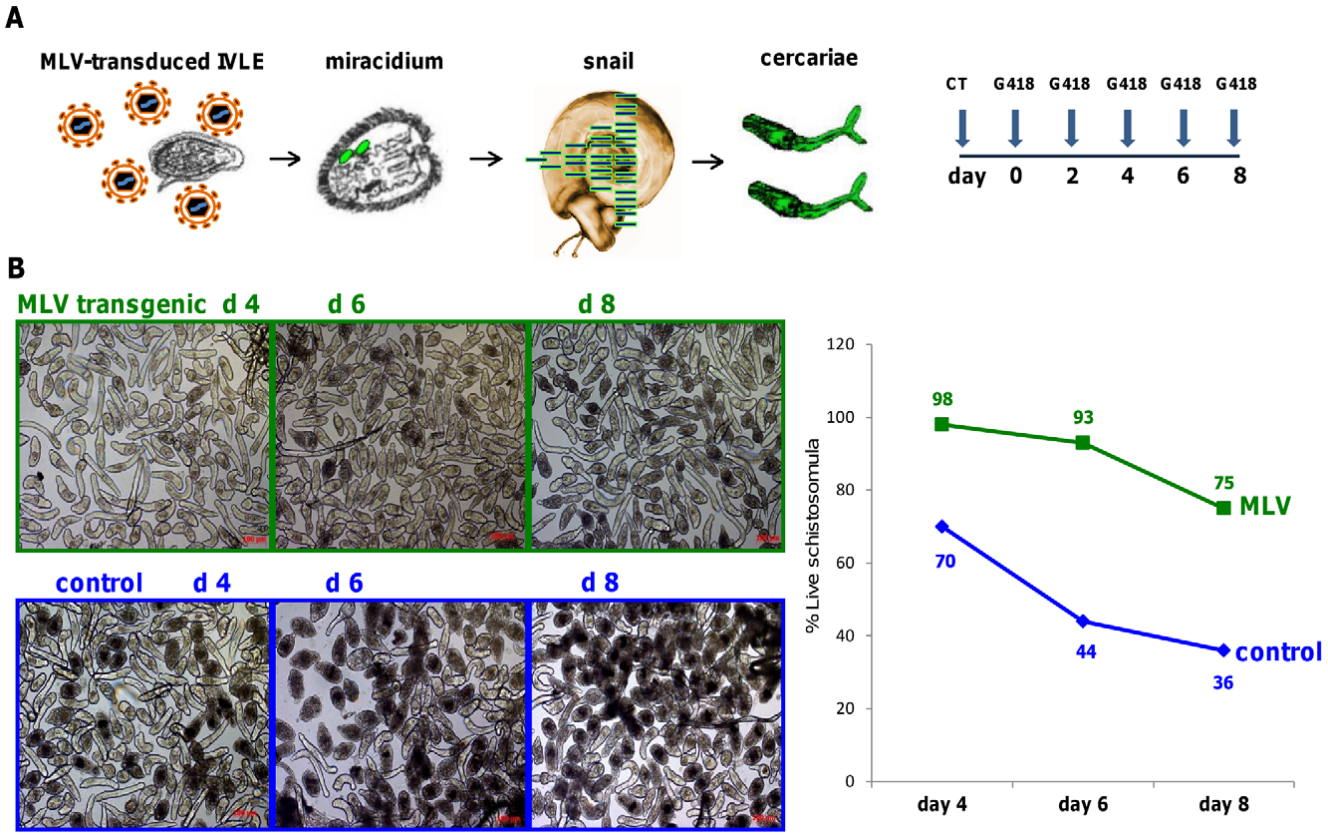
Rescue of transgenic schistosomula in the presence of G418 (geneticin). Panel A: Experimental design. *In vitro* laid eggs (IVLE) were transduced with MLV virions and hatched six days later. The miracidia were employed to infect snails and ∼40 days later the cercariae were collected. The presence of the transgene in the cercarial genomic DNA was verified by qPCR and Illumina sequencing. Schistosomula were obtained by transformation of cercariae (cercarial transformation, CT) and cultured in 250 µg/ml G418 as described [29]. Culture media including G418 were replaced every second day for eight days. Control schistosomula from wild type schistosomes (i.e. non-transgenic) were included. Panel B: Left top; representative micrographs taken on days 4, 6 and 8 of transgenic schistosomula (derived from snails infected with MLV transduced eggs/miracidia) cultured in G418, as indicated. Left bottom; representative micrographs taken by days 4, 6 and 8 of control (i.e. non-transgenic) schistosomula cultured in G418, as indicated. Right; survival of transgenic schistosomules derived from snails infected with MLV transduced eggs/miracidia (green) and control wild type schistosomula (blue), cultured in G418.

Exposing IVLE to MLV lead to transgenic cercariae, as determined by quantitative PCR (qPCR) and high throughput (Illumina) sequencing (Table 1 and above). Given that the transgenic lines carried and expressed the retroviral *neoR* transgene [36], [37] which confers resistance to the aminoglycoside antibiotic G418 ( = geneticin) [29], we examined whether the germline transgenic progeny (schistosomula), a line named IVLE_MLV_001, exhibited resistance to G418 (Figure 4A). Control, non-transgenic schistosomula were killed by 250 µg/ml of G418: for example, 30%, 56% and 64% were dead by days 4, 6 and 8, respectively. By contrast, >98%, 93% and 75% of schistosomules from the germline transgenic population survived at days 4, 6 and 8, respectively (Figure 4B). The findings indicated that the retroviral transgene encoding *neoR* transmitted vertically through the germline, rescued the schistosomules from toxicity of the antibiotic. Finally, in preliminary analysis of three additional lines of transgenic cercariae, lines IVLE_MLV_002, IVLE_MLV_003 and LE_MLV_001, *neoR* transcripts were detected in schistosomules at 48 hours in culture whereas expression was not evident in cercariae of these lines (not shown).

## Discussion

This investigation presents potentially transformative advances for functional genomics of schistosomes and indeed parasitic helminths at large. First, high-throughput sequencing revealed the genomic locations of numerous integrations of MLV and demonstrated that retroviral integration events were widely and randomly distributed along all eight schistosome chromosomes, including the Z/W sex chromosomes of *Schistosoma mansoni*. Thus, MLV randomly mutagenized the genome of developmental stages of schistosomes. Second, retroviral transgenes were transmitted through the asexual developmental cycle of the schistosome in the intermediate host snail, from miracidia to cercariae, confirming germline transgenesis, and subsequently to eggs of the F1 generation. Third, a germline-transmitted transgene, *neoR*, encoding neomycin phosphotransferase permitted schistosomules to survive toxic concentrations of the aminoglycoside antibiotic G418.

Detection of transgenes in cercariae showed that MLV successfully integrated into the genome of eggs, and was maintained and vertically transmitted through the germline of the miracidium, mother sporocyst and daughter sporocyst stages. Daughter sporocysts derive only from the germline of the mother sporocyst, and cercariae from the germ cells of the daughter sporocyst. Consequently, transgenes in cercariae can only have arisen through germline transmission [15]. Of >1,200 authentic integration events mapped to the schistosome chromosomes, more than 900 were recovered from transgenic cercariae. All cells of these cercariae would be expected to carry the retroviral transgene(s) whereas, in the somatic approach, transgenic worms would be constituted of mosaics of transgenic and non-transgenic cells. The MLV constructs employed here encode virions that cannot replicate, hence only the transduced cells and their progeny would carry the transgene.

The pseudotyped MLV virion represents a tractable delivery vector to produce transgenic schistosomes, as demonstrated in studies on integration and activity of MLV-delivered reporter transgenes in *S. mansoni* and *S. japonicum* [32], [34], [36], [38]–[41]. Advantages of this system include autonomy, since the envelope glycoprotein of the vesicular stomatitis virus binds to an uncharacterized receptor on schistosome cells, initiating transduction by the retrovirus, and the ability of retroviral integrase to insert the proviral transgene into schistosome chromosomes. Among developmental stages that have been targeted by MLV [34], [38], [40], the egg and miracidial stages of *S. mansoni* provide access to germ cells (see [15], [34], [39]). Here, two forms of eggs were transduced with MLV - eggs from mouse livers (LE) and *in vitro* laid eggs (IVLE). IVLE provides a compliant developmental stage at which to introduce transgenes into the schistosome germline, since in stages 0 and 1 of the developing egg (staging system of Jurberg et al [42]), cleavage of the zygote has yet to occur [34]. LE – although available in greater numbers than IVLE - range in age and development from newly laid eggs through several weeks, and also include eggs injured by host responses. Transduction by MLV resulted in transgenic cercariae in 10 of 13 experiments with LE, but only in three of 10 with ILVE. However, the copy number of transgenes in genomic DNA isolated from populations of cercariae was ∼20 times higher in larvae originating from MLV transduced IVLE than LE. On the other hand, snails were likely exposed to more LE miracidia, a few to 200 per snail, whereas numbers of IVLE miracidia was likely considerably less – maybe as few as one miracidium per snail. With this regimen, even if the number of transformed miracidia was similar between LE and IVLE, higher numbers of non-transformed larvae may have diluted transgene density in LE-associated cercarial populations, yielding lower transgene copy numbers. To definitively determine whether MLV transduction of IVLE leads to higher transgene density in the schistosome genome, it would be informative to employ identical numbers of IVLE and LE miracidia per snail and compare transgene copy numbers in cercariae. If transduction of IVLE generally leads to higher transgene numbers, this would be advantageous if saturating insertional mutagenesis was the goal. By contrast, transduction of LE may lead generally to lower transgene copy numbers. Approaches targeting LE have apparent advantages since the miracidia more readily infect snails. LE may be preferable where the goal is to express transgenes encoding native or mutant gene products in studies of schistosome gene function. Here, minimizing transgene copy number to avoid non-specific effects e.g. of over expression or mutagenesis may be desirable. In review, whether to target IVLE rather than LE will depend on the goals of the investigation. This is the first report of germline transmission of an integration competent vector in schistosomes and hence opens the door to the development of transgenic lines of schistosomes.

We have begun to establish lines of transgenic schistosomes, and indeed have named the first line IVLE_MLV_001 (this line originated from experiment 9, Table 1B). In early investigation of the durability of the transgenic line and retroviral transgene(s), mice were infected with transgenic cercariae by the percutaneous route. In due course, adult worms were recovered, which released IVLE. Both the *neoR* and *luciferase* transgenes were detected by PCR in genomic DNA from the adults (G0) and from the eggs (F1). Hence, retroviral transgenes were transmitted vertically through the asexual (mitotic) and the sexual (meiotic) reproductive phases of the developmental cycle of *S. mansoni*. It is germane to note that from this transgenic line, monitored so far from G0 eggs to FI eggs, the schistosomes appeared to be biologically fit in that miracidia infected snails, cercariae infected mice, and eggs were passed in mouse feces. Genome instability has been described in schistosomes [43] which may lead to chromosomal rearrangements during mitosis and/or meiosis, and somatic mutations at microsatellite loci may be routine in intra-snail stages [44]. Mitotic recombination leading to loss of transgenes occurs only rarely in model eukaryote species [45], [46]. However, given the transgene copy number in F1 eggs was lower than the parental cercariae, loss of transgenes through genome instability cannot be ruled out. Nonetheless, since the transgene was detected in cercariae derived from MLV-transduced eggs, even if genome rearrangement occurs in the IVLE_MLV_001 line, so far it has not resulted in elimination of the transgene.

In a previous study, a small number of retroviral transgene integration sites (<20 sites) into chromosomes were characterized by employing anchored PCR [36]. This approach constrains detection of integration sites to those in the vicinity of endogenous mobile elements which were targeted with gene specific primers in the anchored PCR [36]. By contrast, high throughput sequencing of randomly sheared genomic DNA (and hence without bias of fragments for PCR anchored regions and/or restriction sites) was utilized here, an approach validated for transposon-based insertional mutagenesis of bacteria [27]. At least 1,248 integration events were mapped within the chromosomes of *S. mansoni*, both from schistosomules and adults (likely retroviral integrations in surface and/or gut cells), and from cercariae derived from virion transduced eggs (a vertical, germline transmission approach). An improved genome sequence [8], [35], advances in qPCR to quantify retroviral transgene copy numbers [40], and construction of Illumina libraries that employed linker mediated PCR products to enrich for retroviral transgene integrations [27], together enabled characterization of numerous transgene insertions in the genome of *S. mansoni*.

Mapping these integrations confirmed that MLV integrated randomly throughout the schistosome genome. Integration events occurred in each of the schistosome chromosomes, including the seven autosomes and both the sex chromosomes Z and W, including Z specific regions. MLV integrations were identified in exons, introns, promoter/5′-UTRs, and intergenic regions, in proportion to the extent of these regions. This distribution is reminiscent of that of avian sarcoma-leukosis virus (ASV) in mammalian chromosomes [47]. Insertion site preferences for retroviruses have been characterized in detail in several systems, primarily in human and mouse cells [48]. In these hosts, MLV integrates throughout the entire genome, but with a bias for transcriptionally active regions, especially 5′-untranslated regions of RNA polymerase II driven genes, CpG islands, DNAse I hypersensitive sites and transcription factor binding sites [49]. HIV-1 also distributes throughout the human genome, preferentially in transcriptional units, but without the positive bias of MLV for promoter regions/transcription start sites [50]. Integration site selection by HIV-1 and HIV-1-based vectors is controlled by the LEDGF/p75 protein [51], [52]. Among other retroviruses, xenotropic murine leukemia virus-related virus exhibits a strong preference for transcription start sites, CpG islands, DNase-hypersensitive sites, and gene-dense regions, chromosomal features associated with structurally open transcription regulatory regions [53]. ASV shows a more random genomic integration pattern, with only weak preference for active genes and none for transcription start regions [47], [50]. In CD4+ T lymphocytes, integration by MLV favors outward-facing major grooves on nucleosome-wrapped DNA, similar to the integration pattern of HIV [54]. The distribution of MLV integrations in the *S. mansoni* genome appeared to be largely random. No bias was observed for MLV to integrate into four genome features examined in detail (intergenic regions, exons, introns and promoter/5′-UTRs) and, moreover, there was no preference apparent for genes predicted to be actively transcribed at the developmental stage during viral transduction. These findings of random integration widely across all eight schistosome chromosomes indicate that MLV has great potential for insertional mutagenesis as a functional genomics approach in this pathogen.

We had observed previously in a small scale study that MLV integrations into schistosome chromosomes were often truncated. The 5′-end of the integrated provirus had lost up to 2 kb, including reporter transgenes [36]. We have now confirmed this phenomenon, but in the ∼300 integrations sampled here, only ∼25% exhibited deletions. (By contrast, the new findings did not confirm presence of a primary sequence motif, gCATcc at the integration site [36] (not shown).) Given that Illumina reads are only ∼75 bp in length and primers targeting internal sites on the LTR of the retrovirus were used to evaluate truncated integrations, many truncated transgenes may not have been detected. This is a technical constraint of the Illumina approach and consequently numbers of truncated transgenes may be underestimated. Truncation events might be evaluated more comprehensively by sequencing approaches that provide longer reads [55]. Truncation of integrated provirus of Human T-Cell Leukemia Virus (HTLV-1), including loss of the 5′-LTR, is known from human tissues where it appears to influence onset of HTLV-1 induced tumors [56]. The unusual behavior of MLV within schistosome chromosomes in terms of absence of integration site preferences along with occasional truncation of provirus may reflect absence or incompatibility of host cofactors in schistosomes that participate in the retroviral developmental cycle. A deeper knowledge of the behavior of MLV in schistosomes could provide leads for novel interventions both for schistosomiasis and/or retroviral infections.

Transgene constructs utilized here included the *neoR* gene that confers resistance to aminoglycoside antibiotics. Schistosomules of *S. mansoni* are sensitive to G418, and *neoR* expression under the control of the 5′-LTR of the MLV retrovirus can confer antibiotic resistance to transgenic schistosomes [29]. We now show that transgenic schistosomules were more resistant to G418 than wild-type schistosomules. Despite occasional truncations in proviral transgenes (Figure S3) [36], this observation indicates that reporter transgenes encoding resistance to G418 are functional. Furthermore, in a discrete analysis of three additional transgenic lines, *neoR* transcripts were detected in schistosomules whereas expression was not evident in cercariae. This predicts that the 5′-LTR promoter of MLV may be inactive in non-mammalian stages of the schistosome. Studies on schistosome development expression of *neoR* driven by the 5′-LTR of MLV should be informative.

The findings revealed wide-scale random insertional mutagenesis of schistosome chromosomes. This is the first report of germline transmission of a transgene in schistosomes, in any platyhelminth or indeed any lophotrochozoan. (This is also the first report to present chromosomal integration site preferences for a retrovirus in any invertebrate.) Establishing systems of conditional transgene expression [31], including model antigens [57] and vaccine candidates [58] in schistosomes will facilitate analysis of pathophysiology of schistosomiasis as well as fundamental aspects of host-parasite relationship including genome methylation and epigenetics [59]. Moreover, transgenic schistosomes expressing antibiotic resistance can be expected to expedite functional genomics based advances with these etiological agents of major neglected tropical diseases.

## Methods

### Ethics statement

Female Swiss-Webster mice infected with the Puerto Rican NMRI strain *S. mansoni* were obtained from the Biomedical Research Institute (BRI) Rockville, Maryland. Adult worms were perfused from these mice at seven weeks after infection [60]. Maintenance of the mice infected with *S. mansoni* at GWU was approved by the GWU Institutional Animal Care and Use Committee of the IACUC of The George Washington University). All procedures employed were consistent with the Guide for the Care and Use of Laboratory Animals.

### Schistosomes

Mice and *B. glabrata* snails infected with the NMRI (Puerto Rican) strain of *S. mansoni* were supplied by Dr. Fred Lewis, BRI. Both adult worms and eggs (LE) were recovered from infected mice [61], using a protocol approved by the Institutional Animal Case and Use Committee of The George Washington University. In addition, eggs laid *in vitro* by female worms were collected [34]. Briefly, after recovery by perfusion from mice, adult worms were washed and transferred into 74-µm diameter mesh Netwell, 6-well plates where they were maintained in culture at 37°C for 48 h [60]. (Eggs laid after the females have been in culture for >48 hours do not develop correctly [33].) The *in vitro* laid eggs (IVLE) from these worms fall through the mesh and collect on the bottom of the culture plate. IVLE were collected and concentrated by filtering media through 8 µm mesh Transwell [34]. Thereafter, IVLE were maintained in schistosomula medium where they develop and mature within 7 days. At that point, they were transferred to sterile water and illuminated with bright light to stimulate hatching. Eggs (∼20%) hatched within 120 minutes, releasing miracidia.

Cercariae released from infected *B. glabrata* snails were mechanically transformed into schistosomula [60]. In brief, cercariae were concentrated by centrifugation (425 g/10 min) and washed once with Dulbecco's modified Eagle's medium (DMEM) supplemented with 200 units/ml of penicillin, 200 µg/ml of streptomycin, 500 ng/ml of amphotericin B and 10 mM HEPES. Cercarial tails were sheared off by 20 passes through 22 gauge emulsifying needles after which schistosomule bodies were isolated from tails by Percoll gradient centrifugation [62]. Schistosomula were washed and cultured in Basch's medium [63] at 37°C under 5% CO_2_.

Female inbred Balb/c mice were infected with ∼200 cercariae of transgenic line IVLE_MLV_001line by the percutaneous route by tail immersion [64]. From day 42 after infection, feces of infected mice were inspected for *S. mansoni* eggs; several voided pellets of feces were re-suspended in PBS and aliquots of the fecal slurry on a microscope slide directly observed with a Zeiss Axio Observer A.1 inverted microscope fitted with a AxioCam ICc3 camera (Zeiss). This microscope system also was used to examine IVLE, LE and other developmental stages, as described [34], [60].

### Culture of schistosomules in the antibiotic G418

*In vitro* laid eggs (IVLE) were transfected with MLV virions, hatched 6 days later, and the resultant miracidia were employed to infect snails. Cercariae released from those snails were collected ∼50 days after infection and presence of retroviral transgenes in the cercariae verified by qPCR and Illumina sequencing. Schistosomula obtained by mechanical transformation of these cercariae were cultured in the aminoglycoside antibiotic G418 ( = geneticin) at 250 µg/ml [29]. Media and G418 were replaced every second day, from days 0 to 8. A control group of non-transduced (wild type) schistosomula was included. At least 100 schistosomules (range, 106–144) per condition were counted, and viability was assessed as described [29].

### Murine leukemia virus, retroviral constructs, transduction of schistosomes

Vesicular stomatitis virus glycoprotein-pseudotyped murine leukemia virus (MLV) virions were produced in GP2-293 cells [36], using pLNHX (Clontech), pLNHX_SmAct-Luc, pLNHX_SmAct-GFP, pLNHX_SLGFP, pLNHX basic, pLNHX_ΔD70 or pLNHX-cHS4 [34], [36], [38]. Viral titers were determined using two complementary approaches; first, a functional assay involving titrating virions on NIH-3T3 cells and, second, real time PCR targeting the retroviral genome (Retro-X qRT-PCR Titration Kit, Clontech) [34], [40]. Schistosomula (∼2–5×10^4^), mixed sex adults, eggs isolated from liver and IVLE of *S. mansoni* were transfected with MLV virions as described [34], [39], [40]. In brief, one to seven days after mechanical transformation of cercariae, schistosomula were cultured in 6 well plates containing one ml of virion preparation with an infectivity of 10^6^–10^7^ colony forming units (CFU)/ml, in the presence of the cationic polymer polybrene. After exposure to virions for 18 hours, culture media were replaced with virion-free media. The following day, retrovirus-exposed schistosomula were harvested, snap frozen and stored at −80°C for downstream analysis. Adult worms were cultured in 24 well plates, in 200 µl of complete medium supplemented with 200 µl of MLV virions (4×10^5^ CFU/ml) and 8 µg/ml polybrene. The worms were washed 18 h later, cultured for a further 24 h, harvested, snap frozen and stored at −80°C.

Eggs from livers of infected mice were cultured in 24 well plates in 500 µl medium containing MLV virions at 10^5^–10^6^ CFU/ml and 8 µg/ml polybrene. Other eggs exposed to the same MLV inoculum were subjected to square wave electroporation [39], and transferred into media containing 8 µg/ml polybrene. Eighteen hours later, eggs were washed to remove unbound virions and polybrene, and cultured for a further 24 h. At this point, eggs were transferred to water, and miracidia released from hatched eggs counted [60]. Miracidia were used to infect *B. glabrata* snails; en masse infection of groups of snails was undertaken using ≤5 to 200 miracidia per snail (Table 1). Other miracidia were snap frozen and stored at −80°C. IVLE were collected from mixed sex adult worms from 0 to 48 h after perfusion of adult worms from mice [34]. IVLE were exposed to virions (10^5^–10^6^ CFU/ml) in 500 µl culture media and 8 µg/ml polybrene for ∼18 h, after which IVLE were washed to remove unbound virions and polybrene and transferred to media without virions. Media were changed every day for five days, until eggs developed to stages 7 and 8 [42]. At this point, eggs were washed with PBS, transferred to sterile water, and induced to hatch by exposure to light [34].

### Detection of provirus and estimation of transgene copy number

Total genomic DNA (gDNA) was isolated from transduced and/or transgenic developmental stages of schistosomes, including mixed sex adult worms, schistosomules, and cercariae, using a kit (E.Z.N.A. SQ Tissue DNA Kit, Omega Bio-tek). Concentrations of gDNAs were determined with a spectrophotometer (NanoDrop 1000). To investigate the presence of proviral transgenes and estimate the transgene copy number by qPCR, primers were designed with Beacon Designer (Premier Biosoft International, Palo Alto, CA) to obtain primer and TaqMan probe sequences targeting the firefly luciferase (*FLuc* from pGL3-Basic, Promega): forward primer: 5′-TGC TCC AAC ACC CCA ACA TC- 3′; reverse primer: 5′- ACT TGA CTG GCG ACG TAA TCC- 3′; probe: 5′-/56-FAM/ACG CAG GTG TCG CAG GTC TTC C/3IABlk_FQ/-3′, and the neomycin phosphotransferase II gene (*neoR*): forward primer, 5′-GGA GAG GCT ATT CGG CTA TGA C-3′; reverse primer, 5′-CGG ACA GGT CGG TCT TGA C-3′; probe, 5′-/56-FAM/CTG CTC TGA TGC CGC CGT GTT CCG/3IABIk_FQ/-3′.

qPCR reactions were performed in triplicate, using 96-well plates, with a denaturation step at 95°C of 3 minutes followed by 40 cycles of 30 sec at 95°C and 30 sec at 55°C, using a thermal cycler (iCycler, Bio-Rad) and a Bio-Rad iQ5 detector to scan the plates in real time. Reactions were carried out in 20 µl volumes with primer-probe sets and Perfecta qPCR FastMix, UNG (Quanta Bioscience, Gaithersburg, MD). Absolute quantification was undertaken using 250 ng of gDNA samples, including non-MLV transduced samples as negative controls, or copy number standards, i.e. 10-fold serial dilutions of pGL3, from 1.93×10^10^ copies to 1.93×10^3^ copies. The exact copy number of each diluted plasmid was calculated through the relationship between the molecular mass of pGL3 and the Avogadro constant, NA. Absolute copy number of the luciferase transgene per ng of schistosome gDNA was estimated by interpolation of the sample PCR signals from a standard curve [65].

### Analysis of transgene expression

Total RNA was extracted from pellets of cercariae and two-day old schistosomules from transgenic lines IVLE_MLV_002, IVLE_MLV_003 and LE_MLV_001 (unpublished). Transgene expression was examined by qRT-PCR, as described [29].

### Illumina libraries

Illumina libraries were constructed as described [66]. In brief, genomic DNAs from schistosomula, adults and cercariae (10–20 µg each), isolated as above, were subjected to ultra-sonication using a model S220 Covaris Adaptive Focused Acoustics instrument (Covaris, Woburn, MA), releasing fragments of ∼200 bp in length. The sheared fragments were modified for ligation by end-repair and adenylation with the NEBNext DNA Sample Prep Reagent Set 1 from New England Biolabs (Ipswich, MA), according to the manufacturer's instructions, but using 1.5× the recommended volumes. Thereafter, the fragments were ligated to double-strand adapters (10-fold excess), formed by annealing oligonucleotides Ind_Ad_T (5′-ACACTCTTTCCCTACACGACGCTCTTCCGATC*T-3′, the asterisk indicates phosphorothioate) and Ind_Ad_B (5′ pGATCGGAAGAGCGGTTCAGCAGGAATGCCGAGACCGATCTC-3′). Approximately one µg of the adaptor-ligated fragments was used to specifically amplify the 5′ or 3′ termini of the MLV retrovirus and retroviral insertion sites. PCRs were performed with Jumpstart Taq polymerase (Sigma-Aldrich, St. Louis, MO). For the first round of the semi-nested PCR, a retrovirus-specific forward primer (Table S1, tab “Primer combinations”, designated “PCR1”), and the reverse primer RInv4.0 (5′ TCCCTACACGACGCTCTTCCGATCT-3′) were used with the program 94°C 2 min, [94°C 20 sec, primer-specific annealing temperature 20 sec, 72°C 40 sec]x18, 72°C 10 min. A second semi-nested PCR was set up with 5 µl of the first PCR as template. The retrovirus-specific primer for this reaction (“PCR2”) contained the Illumina P5 end for attachment to the flow cell, the adapter-specific primer included the Illumina P7 end, and an 8 nt tagging sequence (Table S1). For DNA from cercariae, DNA was subjected to 28 cycles for PCR1, and 16 cycles PCR2. PCR product libraries were quantified by qPCR with standards of known concentration, using primers Syb_FP5 (5′-ATGATACGGCGACCACCGAG-3′) and Syb_RP7 (5′-CAAGCAGAAGACGGCATACGAG-3′). Equal amounts of libraries were pooled where appropriate (the two retrovirus 5′ and 3′ ends were sequenced in separate pools), and size separated on an agarose gel. Fragments of 350 to 450 bp were excised and recovered with QiaExII gel extraction columns (Qiagen) following the manufacturer's instructions, but without heating [66].

The DNA fragment libraries were quantified by qPCR, as above, denatured and sequenced for 76 cycles on paired end flow cells on an Illumina GAII platform (Illumina, Inc., San Diego, CA) using custom sequencing primers (Table S1, tab “Primer combinations”) for the first read, the regular Illumina Read1 primer 5′-ACACTCTTTCCCTACACGACGCTCTTCCGATCT-3′ for Read2, and primer 5′-AGATCGGAAGAGCGTCGTGTAGGGAAAGAGTGT-3′ for the index read. About 6.5 million (M) reads deriving from the 5′-end of the retroviral transgene were detected (6 M, 227 k, and 296 k, from three flowcell lanes), and 16.6 M from the 3′-end (12.6 M, 203 k, 221 k, and 3.59 M from four flowcell lanes). For cercariae, 6.3 M reads were obtained on one lane for the 5′ end, and 1.5 M for one lane of the 3′ end of the transgene.

### Bioinformatics analysis

Sequence reads from the Illumina FASTQ files were be parsed for ∼100% identity (with one mismatch allowed, below) to the last 7 bp of the 5′-end or 3′-end of the retrovirus sequence. Thereafter, matching sequence reads were stripped of this retrovirus tag, converted to Sanger FASTQ format and mapped to the *S. mansoni* genome [8], [35]. After an initial round of quality control (removal of PCR adaptors, vector clipping), the 76 bp Illumina sequences were mapped to the v5.1 *S. mansoni* assembly using SMALT, http://www.sanger.ac.uk/resources/software/smalt/. Sequence matches were considered a genuine integration if (1) the Illumina sequence started with the retrovirus long terminal repeat terminus with at most 1 bp mismatch, (2) the remainder of the sequence uniquely mapped to the reference sequence with at least 40 bp, (3) with a mapping quality ≥30 (corresponding to a 0.1% alignment error rate) and (4) the flanking sequence of the matched region was different to the MLV retrovirus start site by at least 2 bp. Matches that mapped to low complexity regions of the genome were discarded. Multiple matches within 200 bp of each other were classified as one unique match in the genome assembly. We categorized the integration region as exon, intron, intergenic, and promoter/5′-UTR by comparing annotations from GeneDB. Since information on UTRs and/or promoters remains largely unavailable for the majority of genes of *S. mansoni*, we arbitrarily assigned the canonical 3 kb upstream of first exon of each gene as the putative promoter/5′-UTR region.

### Truncated transgenes

To examine the whether truncations occurred in the integrated proviral transgenes, we searched for the presence of integrations in a series of Illumina libraries constructed with eight discrete retroviral specific primers targeting sites at 0, 500, 1,000 and 1,500 bp from both the 5′- and 3′-terminus of the retrovirus (Table S1; Figure S2). These libraries were prepared from gDNAs from schistosomules and adult worms transduced with MLV.

### Data access

Sequence data from this study have been deposited in the European Nucleotide Archive (http://www.ebi.ac.uk/embl) under accession number ERP000379.

## Supporting Information

Figure S1 **Propagation of schistosome transgenic line, termed IVLE_MLV_001.** Panel A: Representative images of eggs in feces voided from mice 55 to 62 days after infection with transgenic cercariae. Panel B: Female worm perfused from mice infected with transgenic cercariae and an *in vitro* laid egg (IVLE). Panel C: Representative image of IVLE released from worms *in vitro* from 0 to 48 hours after perfusion from mice infected with transgenic cercariae. Panel D: Autoradiograph of Southern hybridization of radiolabeled gene probe to PCR products amplified using *luciferase* specific primers. The luciferase transgene was detected in 14 of 14 (100%) adult schistosomes of line IVLE_MLV_001 (lanes 1 to 14) but not in wild type schistosomes (lane 15). *Nco* I-digested plasmid pLNHX-SmAct-Luc (lane 16) was included as positive control for the primers and probe. PCRs targeting the actin gene were positive for all worms, both transgenic and wild type control, confirming integrity of the genomic DNAs (not shown). (See [36] for methods.) Panel E: Luciferase transgene copy number in F1 generation IVLE ascertained by qPCR; control, wild type (non-transgenic) schistosomes. Scale bars: 50 µm in panel A and 200 µm in panels B and C. pi, post infection.
(TIF)

Figure S2 **Primer arrangement for** ***Schistosoma mansoni*** **retrovirus mutagenesis.** Arrows (green for 5′-end libraries, red for 3′-ends) designate binding sites of the primer triplets per library. The top primer of every triplet was used for PCR1, the second for the semi-nested PCR2, the third for the sequencing reaction. Sequences of the primers are provided in Table S1.
(TIF)

Figure S3 **Summary of intact and truncated retroviral transgenes after integration into schistosome chromosomes.** Pie charts representing percentages of intact and truncated integrations of the provirus observed in the 5′-end (MLV 5′-end) and 3′-end (MLV 3′-end) Illumina libraries prepared with genomic DNAs from schistosomules and adult worms transduced with MLV. The libraries were constructed with eight discrete retroviral specific primers targeting sites at 0 (intact integrations), 500 (500 bp from the end), 1,000 (1,000 bp from the end) and 1,500 bp (1,500 bp from the end) from both the 5′- and 3′-terminus of the retrovirus.
(TIF)

Table S1 **Sequences of nucleotide primers used in Illumina sequencing.** The tab “Primer combinations” summarizes which primers were used for the two PCR reactions per library, and which primer for sequencing (Illumina Read 1).
(XLSX)
