# Supplementary figures and images for: Germline Transgenesis and Insertional Mutagenesis in Schistosoma mansoni Mediated by Murine Leukemia Virus
Source: PLoS Pathog. 2012 Jul 26;8(7):e1002820. doi: 10.1371/journal.ppat.1002820 (PMC3406096; doi:10.1371/journal.ppat.1002820)

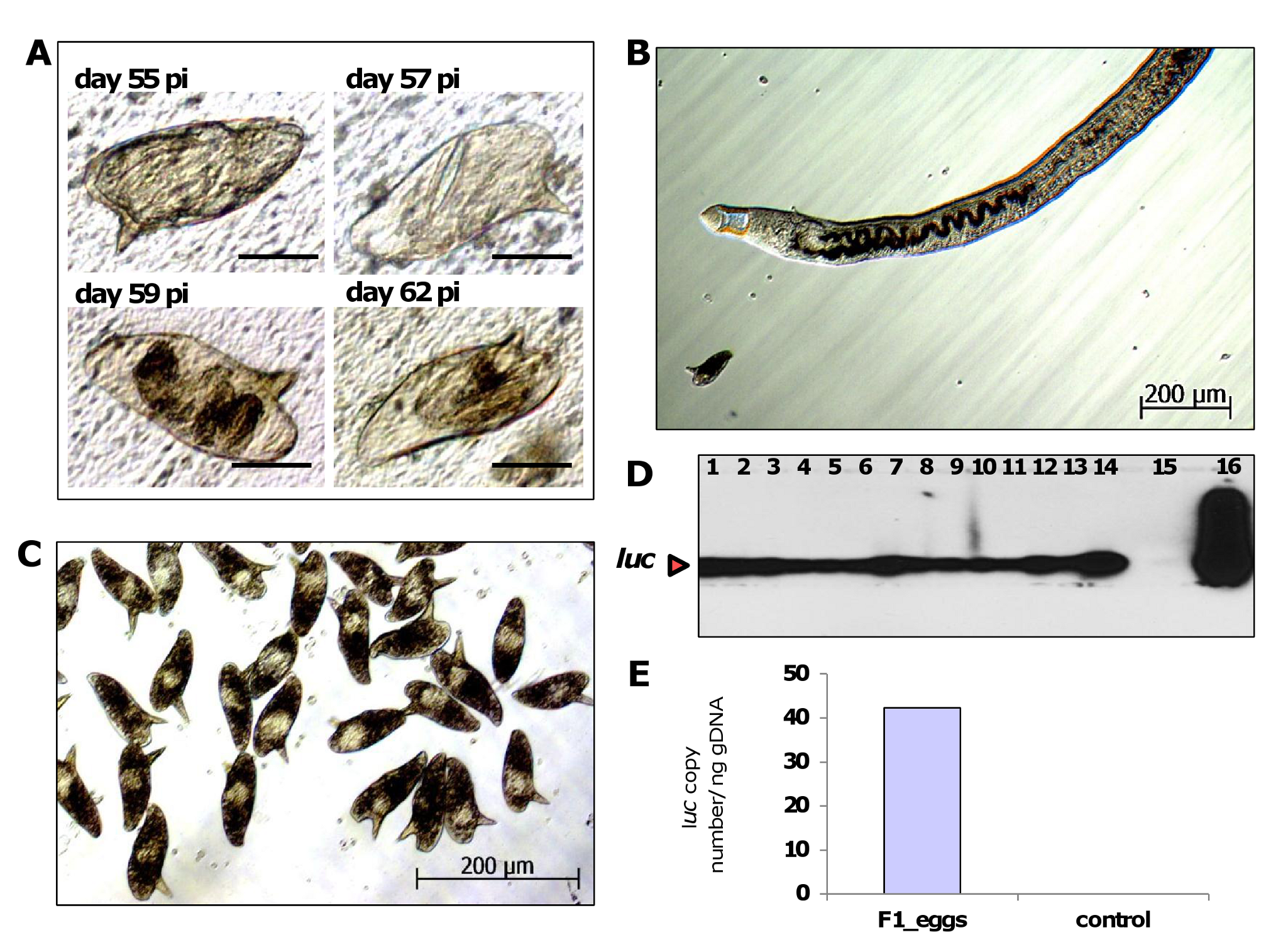

Supplement: Figure S1 — Propagation of schistosome transgenic line, termed IVLE_MLV_001. Panel A: Representative images of eggs in feces voided from mice 55 to 62 days after infection with transgenic cercariae. Panel B: Female worm perfused from mice infected with transgenic cercariae and an in vitro laid egg (IVLE). Panel C: Representative image of IVLE released from worms in vitro from 0 to 48 hours after perfusion from mice infected with transgenic cercariae. Panel D: Autoradiograph of Southern hybridization of radiolabeled gene probe to PCR products amplified using luciferase specific primers. The luciferase transgene was detected in 14 of 14 (100%) adult schistosomes of line IVLE_MLV_001 (lanes 1 to 14) but not in wild type schistosomes (lane 15). Nco I-digested plasmid pLNHX-SmAct-Luc (lane 16) was included as positive control for the primers and probe. PCRs targeting the actin gene were positive for all worms, both transgenic and wild type control, confirming integrity of the genomic DNAs (not shown). (See [36] for methods.) Panel E: Luciferase transgene copy number in F1 generation IVLE ascertained by qPCR; control, wild type (non-transgenic) schistosomes. Scale bars: 50 µm in panel A and 200 µm in panels B and C. pi, post infection. (TIF) [file ppat.1002820.s001.tif]

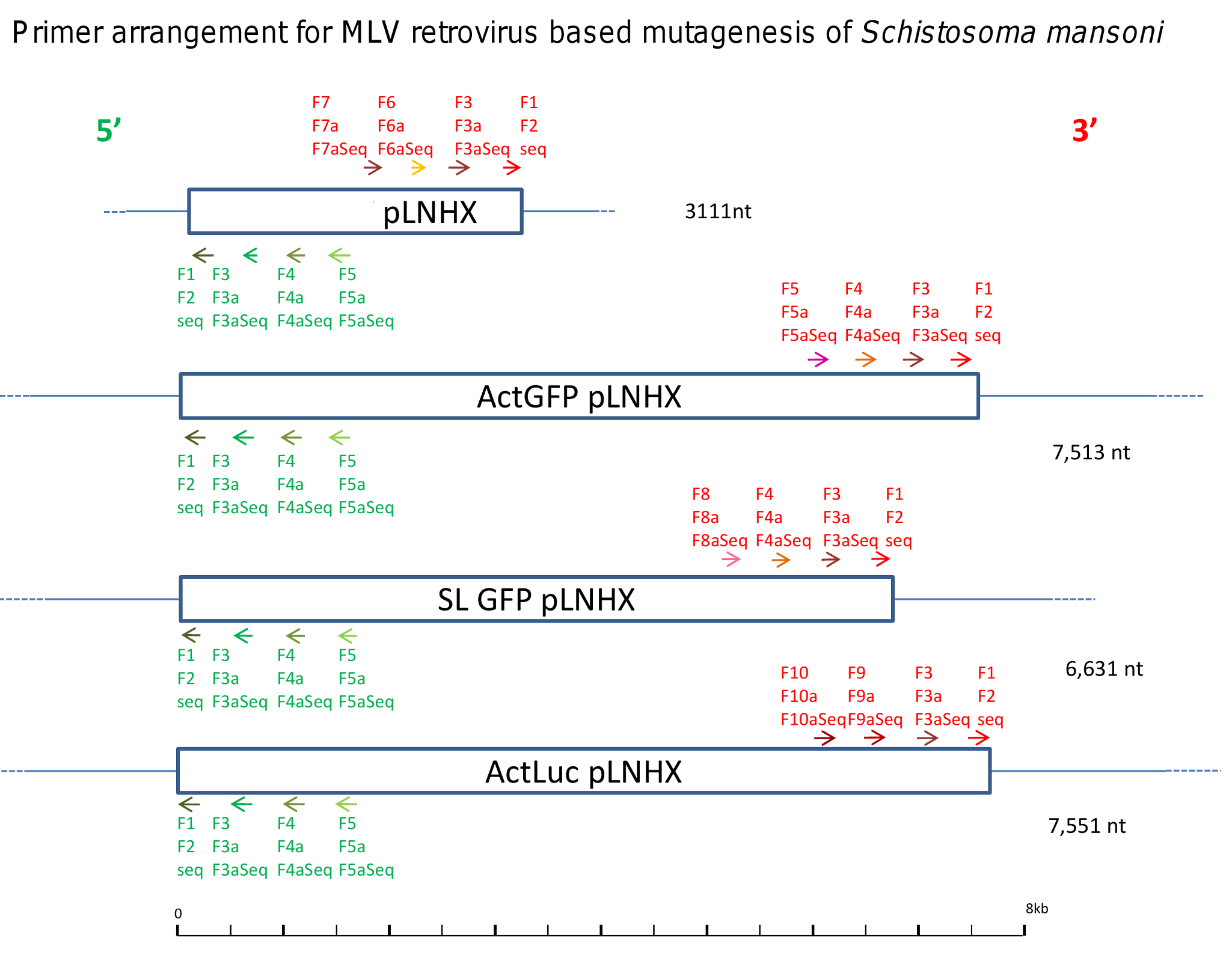

Supplement: Figure S2 — Primer arrangement for Schistosoma mansoni retrovirus mutagenesis. Arrows (green for 5′-end libraries, red for 3′-ends) designate binding sites of the primer triplets per library. The top primer of every triplet was used for PCR1, the second for the semi-nested PCR2, the third for the sequencing reaction. Sequences of the primers are provided in Table S1. (TIF) [file ppat.1002820.s002.tif]

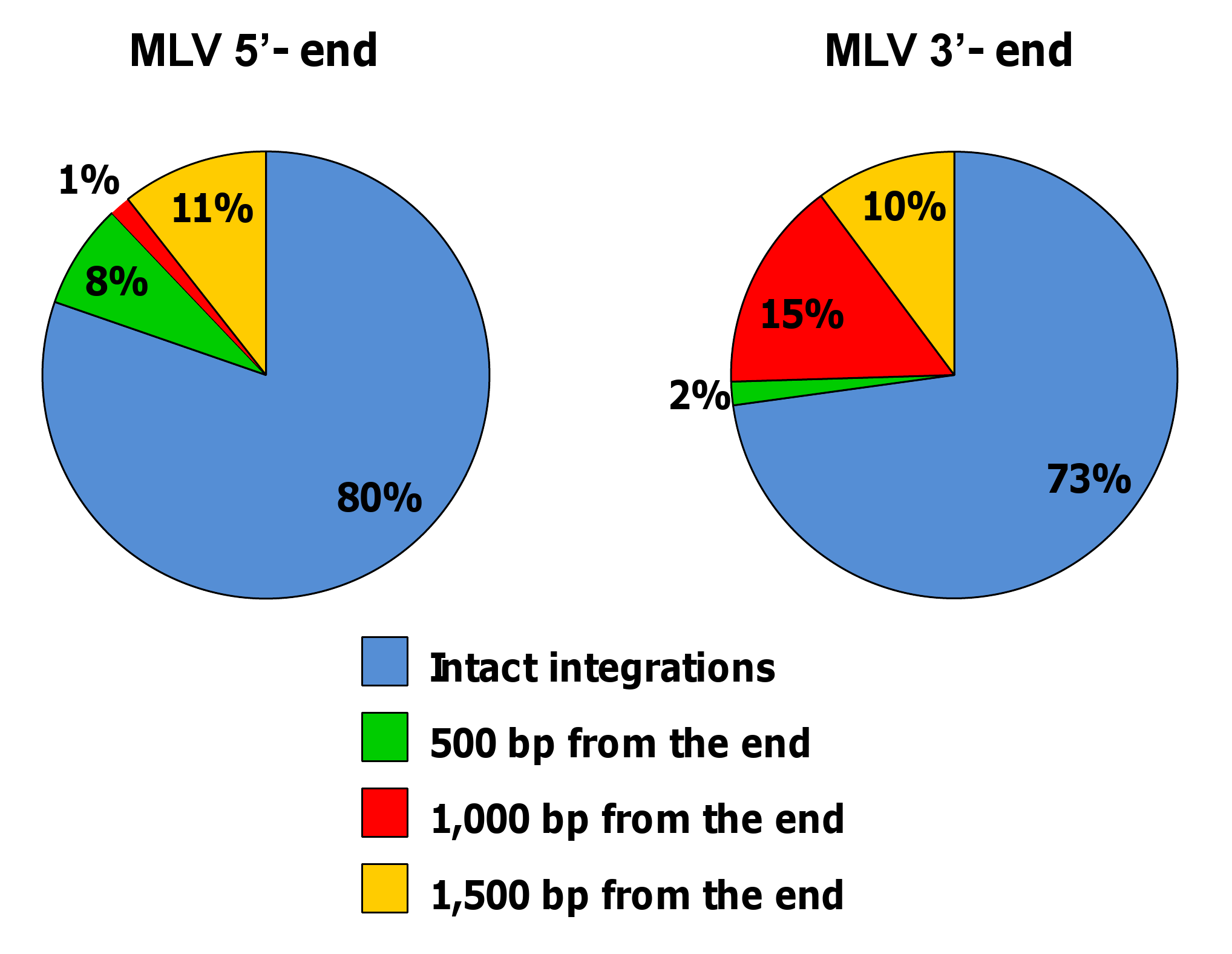

Supplement: Figure S3 — Summary of intact and truncated retroviral transgenes after integration into schistosome chromosomes. Pie charts representing percentages of intact and truncated integrations of the provirus observed in the 5′-end (MLV 5′-end) and 3′-end (MLV 3′-end) Illumina libraries prepared with genomic DNAs from schistosomules and adult worms transduced with MLV. The libraries were constructed with eight discrete retroviral specific primers targeting sites at 0 (intact integrations), 500 (500 bp from the end), 1,000 (1,000 bp from the end) and 1,500 bp (1,500 bp from the end) from both the 5′- and 3′-terminus of the retrovirus. (TIF) [file ppat.1002820.s003.tif]
